# Organic Contaminant Biodegradation by Oxidoreductase Enzymes in Wastewater Treatment

**DOI:** 10.3390/microorganisms8010122

**Published:** 2020-01-16

**Authors:** Edward A. Barber, Ziyi Liu, Stephen R. Smith

**Affiliations:** Department of Civil and Environmental Engineering, Imperial College London, South Kensington Campus, London SW7 2AZ, UK; edward.barber18@imperial.ac.uk (E.A.B.); ziyi.liu15@imperial.ac.uk (Z.L.)

**Keywords:** organic contaminant, redox potential, oxidoreductase enzymes, enzymatic degradation

## Abstract

Organic contaminants (OCs), such as pharmaceuticals, personal care products, flame retardants, and plasticisers, are societally ubiquitous, environmentally hazardous, and structurally diverse chemical compounds whose recalcitrance to conventional wastewater treatment necessitates the development of more effective remedial alternatives. The engineered application of ligninolytic oxidoreductase fungal enzymes, principally white-rot laccase, lignin peroxidase, and manganese peroxidase, has been identified as a particularly promising approach for OC remediation due to their strong oxidative power, broad substrate specificity, low energy consumption, environmental benignity, and cultivability from lignocellulosic waste. By applying an understanding of the mechanisms by which substrate properties influence enzyme activity, a set of semi-quantitative physicochemical criteria (redox potential, hydrophobicity, steric bulk and pKa) was formulated, against which the oxidoreductase degradation susceptibility of twenty-five representative OCs was assessed. Ionisable, compact, and electron donating group (EDG) rich pharmaceuticals and antibiotics were judged the most susceptible, whilst hydrophilic, bulky, and electron withdrawing group (EWG) rich polyhalogenated compounds were judged the least susceptible. OC susceptibility scores were in general agreement with the removal rates reported for experimental oxidoreductase treatments (R^2^ = 0.60). Based on this fundamental knowledge, and recent developments in enzyme immobilisation techniques, microbiological enzymic treatment strategies are proposed to formulate a new generation of biological wastewater treatment processes for the biodegradation of environmentally challenging OC compounds.

## 1. Introduction

Wastewater treatment plant (WWTP) effluents and biosolids are regularly found to contain a wide variety of heterogeneously structured novel micropollutants as chemically engineered production of anthropogenic compounds continues to diversify. Environmental organic contaminants (OCs) can be broadly characterised by their prevalence as ubiquitous necessities (e.g., antibiotics, pharmaceuticals, personal care products, hormones, plasticisers, flame retardants, surfactants, biocides, and preservatives), their recalcitrance to conventional wastewater treatment and natural degradation processes, their environmental persistence and bioaccumulation, their ubiquity at analytically challenging yet ecologically harmful ppb/ppt environmental concentrations, and their general lack of current source control legislation [1]. Importantly, whilst the environmental impacts of OC emission include endocrine disruption, food-web accumulation, ecological toxicity/mutagenicity/teratogenicity, and antibiotic resistance dissemination, the continuous discharge of OCs in wastewater effluent allows for even the least persistent OCs to potentially impart some degree of ecological harm upon receiving waters [2]. Thus, the development of effective, systematic, and economically and environmentally sustainable techniques that facilitate OC removal within the confines of WWTPs is urgently needed.

Recently, the application of isolated enzymes has gathered particularly significant attention for OC degradation [3]. Enzymatic treatment can have higher specificity for very low concentration and highly recalcitrant OCs and it produces less harmful by-products as compared to other physicochemical technologies, such as dense membrane filtration, advanced oxidation, and granular activated carbon adsorption [4,5]. Fungi oxidoreductase enzymes, including white-rot laccase, lignin peroxidase (LiP), and manganese peroxidase (MnP), which are synergistically employed to oxidatively and non-selectively cleave the resilient polyphenol structures of lignin during the decomposition of wood, distinguish themselves as promising candidates to remove OCs. For example, Alharbi et al. found that 5 mg/L of diclofenac, trimethoprim, carbamazepine, and sulfamethoxazole can be effectively degraded by laccase from *Trametes versicolor*, with the efficiency reaching 100%, 95%, 85%, and 56%, respectively [6]. Singh et al. reported that up to 75% of another OC, triclosan (1.5–0.375 mg/L), can be degraded by the free laccase produced from the same species of bacteria [7]. Similarly, Lip and MnP were also reported to be capable of degrading various phenolic and/or nonphenolic compounds under certain conditions [8,9,10,11].

The reaction of these oxidoreductase enzymes follows a radical reaction mechanism, driven by the redox potential differential between the enzyme active site and substrate. Laccase catalysis, for example, is initialised by a cycle of four single outer-sphere electron transfers between four phenolic substrate molecules, being sequentially bound at the primary electron accepting active site (with redox potential at 0.43–0.78 V [12]), which results in substrate oxidation and the generation of polymerisation prone phenoxy free radicals. Phenolic and aromatic compounds, which usually show relatively low redox potential, can be non-specifically decomposed by laccase, Lip, and MnP. Moreover, these enzymes also have ability to expand their specificity towards other non-phenolic and non-aromatic substrates (with redox potentials of up to 1.5V under certain circumstances [13,14]).

However, future deployment of enzymatic treatment of OCs is strongly dependent on the ability to rapidly screen enzymes possessing specificity to the contaminants of concern [15]. This in itself must be founded upon an improved understanding of the factors controlling the susceptibility of a compound to enzymatic catalysis.

Currently, no established and rational technique exists, by which the susceptibility of OCs to enzymatic degradation can be systematically predicted, a fact that restricts the rationalisation of oxidoreductase treatment process design. This study aimed to construct a framework for assessing the susceptibility of OCs to fungal enzyme degradation, founded upon an understanding of the interrelationships between the physicochemical properties of the contaminant, the catalytic mechanism of the enzyme, and the conditions of the environment, and then apply this to recommend key criteria to consider in the design of enzymic treatment systems for OC removal from wastewater.

## 2. Materials and Methods

### 2.1. Shortlist of Target OCs for Assessment

Twenty-five representative contaminants (Table 1) were shortlisted to undergo detailed enzymatic susceptibility assessment. Each compound was selected due to it possessing a combination of a high detection frequency in wastewater/biosolids, a low average wastewater removal percentage relative to other compounds of the same subgroup, and a degree of environmental persistence, bioaccumulation and/or toxicity.

### 2.2. Criterion Selection and Its Rationale

Four physicochemical properties of contaminants that affect their suitability as fungal enzyme substrates are considered as criteria for the assessment process and are described, as follows.

• Criterion 1 (C1): Net electron donating group density

As the one-electron oxidation reactions oxidoreductases employ initialise substrate transformation are driven by the redox potential differential (∆E) between the enzyme and its substrate, substrate molecules with lower redox potentials, weaker electron affinities, and, thus, greater electron transfer tendencies will be more susceptible to enzymatic oxidation, and subsequent transformation/removal [16]. Unfortunately, the structural density of electron donating groups (EDG) and electron withdrawing groups (EWG) was adopted as a proxy for redox potential, as the availability of redox potential data for the shortlisted contaminants is extremely limited. Here, the term: *Net Electron Donating Group Density*, represents the presence, balance, and strength of donating and withdrawing groups within a given contaminant.

• Criterion 2 (C2): Hydrophobicity

Hydrophobicity represents the tendency of a compound to partition and sorb from the aqueous phase into the nonaqueous phase [17]. Hydrophobicity is known to heavily impact upon the removal rates that were observed in wastewater treatment processes [18,19] and also upon the susceptibility of contaminants to purified enzyme degradation [20,21].

• Criterion 3 (C3): Steric hindrance

For substrates only containing small substituents (-OH, -CH_3_, -OCH_3_, C_2_H_5_, etc.), electron donating/withdrawing strength dominates enzyme activity determination. However, for compounds possessing longer chained substituents, the enzyme-substrate (E-S) complex formation at the active site might be severely interfered via steric hindrance effects [22,23].

• Criterion 4 (C4): pKa

Some studies [24,25] showed that compounds with lower pKa values display faster oxidation rates under physiological pH conditions and micropollutants can be more readily degraded by oxidoreductase at a pH that is higher than its pKa.

### 2.3. Criteria Weighting

The weightings of the criteria were determined by considering the relative ability of each criterion to influence the enzymatic oxidation rate of the target OCs. Based on the supporting literature available, the relative importance ranking of each criterion was listed as:

(1) Net electron donating group density [16,21,23,26,27,28];
(2) Hydrophobicity [20,21] and Steric Hindrance [22,23]; and,
(3) pKa [24,25].

As such, the criteria weightings were set to 9-3-3-1 for net electron donating group density, hydrophobicity, steric hindrance, and pKa, respectively.

### 2.4. Scoring for Each Criteria

#### 2.4.1. Criterion 1: Net Electron Donating Group Density

To produce a value of C1, the following methodology was adopted:

- Firstly, each donating and withdrawing substituent within the molecular structure of the shortlisted contaminants was identified and then assigned a score of 3, 2, or 1, relative to its classification as a strong, medium, or weak group, respectively (Table 2).
- The donating and withdrawing group scores of each contaminant were separately tallied and the difference between these summations calculated.
- This difference was then divided by the contaminants molecular weight before being multiplied by 100 to provide a final normalised density-based parameter with units of net donating group score per 100 g/mol.

Overall, the contaminants with a final net electron donating group density value >1 (strong donating group dominance), 0–1 (slight/moderate donating group dominance), or <0 (withdrawing group dominance) were assigned a susceptibility score of 9, 6, or 3, respectively. Contaminants only containing withdrawing groups were assigned a score of 0. However, it must be clarified that, as donating and withdrawing substituents influence redox potential via electron density modification of the aromatic moiety to which they are attached, only the redox potential of aromatic compounds is influenced by their presence. The functional groups that are attached to aliphatic molecules are, therefore, considered to have no positive impact on oxidoreductase susceptibility in this criterion category and, as such, aliphatic contaminants were assigned a net electron donating group density susceptibility score of 0.

#### 2.4.2. Criterion 2: Hydrophobicity

Hydrophobicity of a substance is quantified by the log octanol-water partition coefficient (Log K_ow_). The hydrophobic nature of a compound can be broadly classified, as follows: Log K_ow_ < 2.5 = low hydrophobicity, Log K_ow_ 2.5–4 = moderate hydrophobicity, and Log K_ow_ > 4 = high hydrophobicity [29]. As such, shortlisted contaminants possessing high, moderate, and low log K_ow_ values were assigned susceptibility scores of 3, 2, and 1, respectively.

#### 2.4.3. Criterion 3: Steric Bulk

The steric bulk of a substituent is numerically represented by its A-value. Highly, moderately, and minimally bulky substituents were defined as those with A-values >3, between 3–1 and <1, respectively (Table 2). Contaminants with high, medium, and low steric bulks were, respectively, assigned susceptibility scores of 1, 2, and 3, respectively.

#### 2.4.4. Criterion 4: pKa

Shortlisted contaminants possessing pKa values that were above and below the typical pH of wastewater (pH 7.5) were assigned susceptibility scores of −1 and 1, respectively. The score of contaminants that was devoid of ionisable functional groups was left unchanged.

### 2.5. Data Collection and Analysis

For assessing the susceptibility of the OCs, Appendix A (Table A1) presents the detailed breakdown of EDG/EWG content, log K_ow_, steric bulk, and pKa data used to derive susceptibility scores for each shortlisted contaminant. Relevance analysis was carried out by statistical linear regression.

## 3. Results and Discussion

### 3.1. Susceptibility Assessment Results

**Figure 1:**
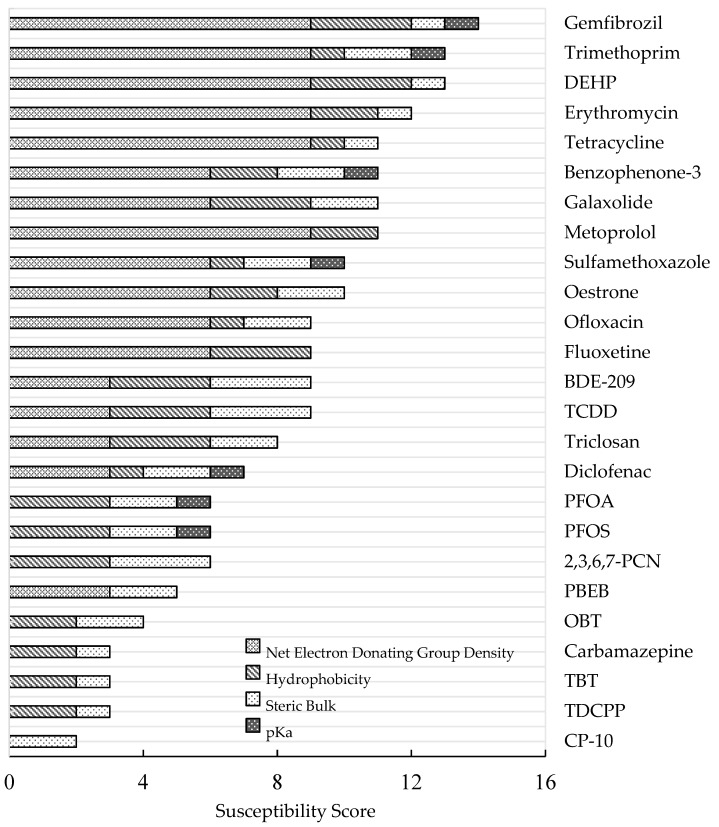
Shortlisted OCs in order of their assessed susceptibility to oxidoreductase degradation. The breakdown of individual criteria scores is represented by patterned bars.

Figure 1 presents the shortlisted contaminants in order of their assessed susceptibility to oxidoreductase degradation within a wastewater treatment context. The higher the total susceptibility score, the greater the predicted susceptibility to oxidoreductase degradation.

From a possible total susceptibility score of 16, Gemfibrozil, a lipid regulating pharmaceutical, scored the highest (14/16), whilst CP-10, which is a short-chained halogenated paraffin/polychlorinated naphthalene, scored the lowest (2/16). The ten most susceptible contaminants comprised four non-halogenated antibiotics (trimethoprim, erythromycin, tetracycline, and sulfamethoxazole), five non-halogenated pharmaceuticals/personal care products (gemfibrozil, metoprolol, benzophenone-3, galaxolide, and oestrone), and one non-halogenated plasticiser (DEHP). Conversely, the fifteen least susceptible contaminants comprised two halogenated surfactants (PFOA, PFOS), four halogenated flame retardants (PBEB, TDCPP, BDE-209, and 2,3,6,7-PCN), one halogenated plasticiser (CP-10), one halogenated antibiotic (ofloxacin), three pharmaceuticals (carbamazepine, diclofenac and fluoxetine), three biocides (OBT, TBT, triclosan), and one combustion by-product (TCDD).

Overall, the halogenated contaminants possessed, on average, a 32% lower susceptibility score (6.6/16) than non-halogenated compounds (9.7/16). This is consistent with the fact that the halogen (-X) groups are electron withdrawing groups; if not balanced with other electron donating groups within the molecule, they tend to increase the redox potential and electrophilicity of a compound by withdrawing electron density away from the aromatic ring [16]. The resulting electron sufficiency of the aromatic moiety decreases the compounds’ susceptibility to oxidative catabolism.

### 3.2. Comparison between the Susceptibility Scores of OCs and Their Removal by Oxidoreductase Treatment

**Figure 2:**
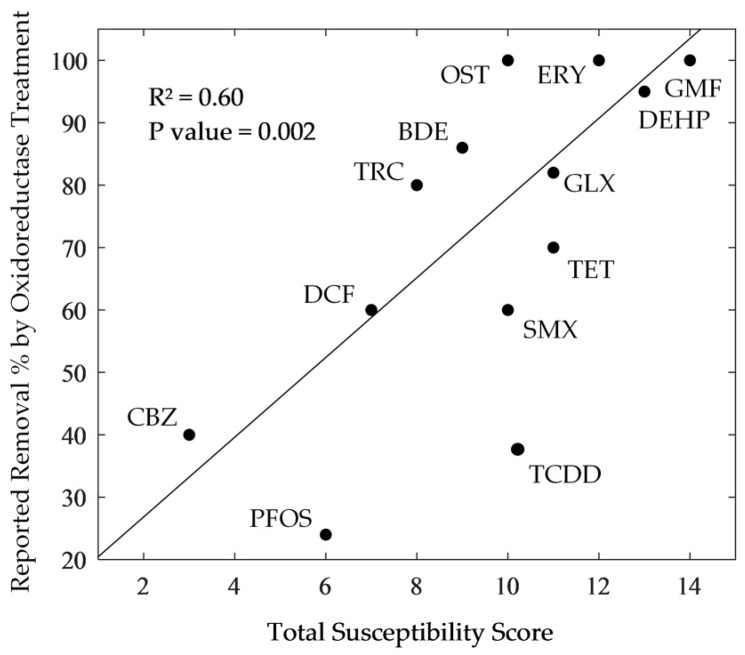
Correlation between total OC susceptibility scores and reported oxidoreductase treatment strategy removal rates (abbreviations were defined in Table 1).

The results of the susceptibility assessment were compared with the published studies of the removal rates that were attained by various oxidoreductase treatment strategies (Figure 2). Linear regression analysis of the relationship showed that the degradation potential of a diverse range of OCs showed good agreement with the susceptibility scores for the compounds and gave an R^2^ = 0.60 (*p* value = 0.002), providing confidence in the assessment framework to predict the susceptibility of OCs to oxidoreductase degradation.

Carbamazepine received a relatively low susceptibility score (3/16) due to its moderate hydrophobicity, high pKa, moderate steric bulk, and lack of EDGs, which is strongly supported by reports of enzymatic recalcitrance. A GAC-immobilised *Aspergillus oryzae* laccase, for example, was reported to achieve 40% carbamazepine removal when compared to 60%, 60%, and 98% for diclofenac (7/16), sulfamethoxazole (10/16), and bisphenol-A, respectively [4]. Similarly, whilst carbamazepine was reported to be negligibly removed by crude laccase (5–37%), LiP (< 10%) and MnP (14–20%), oestrone (10/16) showed much greater degradability under the same treatment conditions (70–100%, 60%, and 99%) [33]. Furthermore, *Tinea versicolor* laccase has been reported to achieve 82% removal of Galaxolide (11/16) [34] and 100% removal of gemfibrozil (14/16) [35], whilst tetracycline (11/16) has been reported to undergo 70% and 72% removal by magnetically crosslinked *Cerrena* laccase [36] and crude MnP [3], respectively. Moreover, *Ganoderma lucidum* laccase, after one day of incubation, was able to degrade 95% of DEHP (13/16) [37], whilst *Pleurotus ostreatus* laccase, after 36 days of incubation, could only degrade 24% of PFOA (6/16) [38]. Finally, removal efficiencies of 43% for carbamazepine (3/16), 64% for diclofenac (7/16), 86% for BDE-209 (9/16), 100% for sulfamethoxazole (10/16), 100% for oestrone (10/16), 100% for benzophenone-3 (11/16), and 100% for erythromycin (12/16) have been reported for the whole-cell *T. versicolor* treatment of thermally dried sewage sludge [39].

However, for certain shortlisted contaminants, susceptibility scores and experimental removal reports did not always fully agree. The oxidoreductase degradation of triclosan, in particular, is more efficient (70–90%) than is expected from its susceptibility score (8/16) [40]. Triclosan is, however, the only shortlisted OC to bear a combination of halogen and phenolic substituents and, as such, the poor association between susceptibility score and the removal rate could arise from the idiosyncratic suppression of halogenic electron withdrawing effects by the donating and polymerisation inducing phenolic substituent [41]. More generally, such inconsistencies might be explained by the non-incorporation of additional known (e.g., reorganisation energy [42]) and ionisation potential [3] and unknown susceptibility criteria.

Furthermore, low susceptibility OCs, such as carbamazepine (3/16), PFOS (6/16), PFOA (6/16), Triclosan (8/16), BDE-209 (9/16), and TCDD (9/16) can still be degraded by the oxidoreductase enzymes under certain conditions, since the enzymes have the ability to expand their specificity towards low susceptibility OCs (e.g., non-phenolic and non-aromatic substrates). Many researchers have reported that the degradation of lower scoring OCs is more strongly predicated in the presence of redox mediators and other complimentary chemical constituents. With the addition of the “redox mediators”, i.e., small diffusible redox-active phenolic co-substrates, such as 2,2′-azinobis(3-ethylbenzthiazoline-6-sulfonate) (ABTS) [43], 1-hydroxy-benzotriazole (HBT) [38,44], and p-coumaric acid (PCA) [45], free radicals generated from the oxidation of these redox mediators can non-specifically shuttle electrons towards, and abiotically oxidise, nearby non-phenolic molecules [21].

### 3.3. Susceptibility Assessment Derived Design Recommendations

#### 3.3.1. Enzyme Reactors and Enzyme Stabilisation

Oxidoreductase enzymes have been applied experimentally within a wide array of engineered systems. Different types of enzymatic reactor have been reported, including: aqueous-phase orientated batch or continuous stirred tank, packed bed, fluidised bed, suspended nanoparticle, hollow fibre microfilter and hybrid membrane-nanoparticle suspension bioreactor systems, as well as solid-phase centric bioslurry and biopile technologies [33,39,46]. However, high rates of pH/thermal enzyme denaturation and low rates of enzyme retention, recovery, and reusability challenge the contaminant removal efficiency and the economic feasibility of enzymatic wastewater treatment [47]. Therefore, the stabilisation of the enzymes is necessary for practical implementation of enzymic treatment. Crucially, immobilisation facilitates the combinatory aggregation or successive application of disparate enzymes, which, in turn, provides the versatility that is required to treat complex pollutant mixtures under fluctuating or poorly defined conditions, which is often the case in wastewater matrices [46]. Conventional immobilisation of enzymes onto solid-supports/carriers (e.g., mesoporous silica and organic gels) by physisorption, entrapment, or encapsulation is regularly reported to reduce enzyme washout from continuous processes, widen enzymatic temperature and pH stability ranges through intermolecular stabilising forces, increase substrate availability by consolidative adsorption, and provide protection from inhibitory molecules [48,49,50]. Recently, an alternative, carrier-free immobilisation technique has been developed to stabilise the enzymes by moderate, covalent bonding with a cross-linker. This immobilisation approach reduces the activity losses that are associated with the inaccessibility of enzymes situated deep within the pores of conventional solid-supports, and it has shown potential for producing enzymatic microreactors to remove organic contaminants from wastewater [51]. For example, Lai et al. prepared nanotubes with laccases that were cross-linked to the surface to remove azo dyes. It was found that a decolourisation rate of 74–96% was possible and 90% of the initial decolourisation rate of the reactor was maintained after 10 sequential batch reactions [52]. Similar results were reported in [53] while using enzymatic membrane reactors that were produced by cross-linking. Lassouane et al. [54] also found that crosslinking of laccases prior to a conventional entrapment method increased the immobilisation efficiency by 30%, and that more than 99% of the contaminant, Bisphenol A, was removed from aqueous solution by cross-linked enzymes.

#### 3.3.2. Optimisation of Enzymatic Treatment

The susceptibility assessment results that are presented here provide a rationale towards establishing potential process design recommendations for the enzymatic treatment of OCs: the treatment performance might be optimised by selecting and manipulating enzymes and wastewater conditions to exploit the fundamental OC susceptibility relationships, as follows.

- Redox Potential: If the typical OC content of a given wastewater stream can be established, and the redox potential of these OCs approximated from EDG/EWG analysis, an enzyme, or redox mediator can be selected that delivers the redox potential that is required for oxidation. The treatment of polyhalogenated OCs in wastewater, for example, is more likely to require the application of lignin peroxidase (1.0–1.2 V) over laccase (0.3–0.8 V), or ABTS (0.68 V) over syringaldehyde (0.51 V) [55], when compared to a predominantly antibiotic/pharmaceutical contaminated influent.
- Steric Bulk/Hindrance: Apart from stabilising the enzymes, the immobilisation techniques were also reported to minimise the steric hindrance of E-S complex formation. PEG, for example, can be employed as a flexible spacer that tethers and non-directly spatially disseminates enzymes across some solid-supports [56]; employment of these flexibly spaced enzymes in a treatment process may orientate certain subunits of OCs towards the enzymes active site in a more favourable way.
- pKa: If enzymatic stability ranges allow, the treatment unit should be operated at a pH that maximises the deprotonation and radicalisation potential of contaminant substituents. For example, an antibiotic and pharmaceutical rich hospital wastewater effluent could be treated by a laccase reactor (pH stability 2–10) at pH ~ 9 to ensure the deprotonation and enhanced susceptibility of sulfamethoxazole (pKa 5.81), trimethoprim (pKa 7.20), erythromycin (pKa 8.90), ofloxacin (pKa 7.65), diclofenac (pKa 4.20), gemfibrozil (pKa 4.70), and fluoxetine (pKa 8.70).

## 4. Conclusions

Twenty-five representative OCs were shortlisted for susceptibility assessment to degradation by lignin degrading fungal enzymes. The physicochemical properties that determine the ability of OCs to act as fungal oxidoreductase substrates, namely redox potential, hydrophobicity, steric bulk, and pKa, were translated into semi-quantitative assessment criteria. The susceptibility scores suggested that oxidoreductase treatment systems are the most effective at degrading EDG-rich, hydrophobic, compact, and ionisable aromatic contaminants, e.g., oestrone, and least effective at degrading EWG-rich, hydrophilic, bulky, and non-ionisable aliphatic contaminants, e.g., PFOS.

The assessment framework successfully incorporated the major criteria that control the efficacy of oxidoreductase OC degradation relative to the reported oxidoreductase treatment removals (R^2^ = 0.60, *p* value = 0.002). Although susceptibility scores and oxidoreductase treatment removal rates largely agreed, the susceptibility of triclosan was underestimated. The accuracy of susceptibility scores may be improved by incorporating additional criteria, or by calibrating criteria weightings by experimentally determining the relative importance of each physicochemical property.

As far as we know, this study is the first to construct a theoretical framework for predicting contaminant susceptibility to oxidoreductase degradation. As such, it has demonstrated the potential for, and practical utility of, theoretically linking the fundamental characteristics of disparate molecules towards deriving a value that broadly signifies the degree to which they will interact. Reasonably, this concept could be adapted for assessing the susceptibility of the contaminants to other treatment processes e.g., advanced oxidation, etc.

Overall, as novel contaminants continue to emerge, the swift and strategic design and deployment of targeted wastewater treatments will become increasingly necessary, this will be more effective if based on theoretical, fundamental assessment frameworks that are similar to that proposed here, to provide rapid and reliable estimations of compound susceptibility and process efficacy. Ultimately, frameworks that predict the performance of oxidoreductase systems could, if developed alongside systems for improving mass enzyme producibility and immobilisation, make the application of fungal enzymes for sustainable wastewater treatment feasible.

## Figures and Tables

**Table 1:** Shortlisted chemicals and their abbreviations.

Chemical	Abbreviation	Chemical	Abbreviation
Sulfamethoxazole	SMX	Triclosan	TRC
Erythromycin	ERY	3,3′,4,4′,5,5′,6,6′-decabromodiphenyl ether	BDE-209
Tetracycline	TET	Pentabromoethylbenzene	PBEB
Ofloxacin	OFL	Tris(1,3-dichloroisopropyl) phosphate	TDCPP
Trimethoprim	TMP	Diethylhexyl phthalate	DEHP
Diclofenac	DCF	2-Hydroxybenzothiazole	OBT
Carbamazepine	CBZ	Tributyltin	TBT
Metoprolol	MTP	Perfluorooctanoic acid	PFOA
Gemfibrozil	GMF	Perfluorooctanesulfonic acid	PFOS
Benzophenone-3	BZP	Tetrachlorodibenzo-p-dioxin	TCDD
Fluoxetine	FLX	2,3,6,7-Pentachloronaphthalene	2,3,6,7-PCN
Oestrone	OST	2,3,4,6,7,8-Hexachlorodecane	CP-10
Galaxolide	GLX		

**Table 2:** Electron donating/withdrawing strength and steric bulk of shortlisted organic contaminant (OC) substituents.

Substituent	Electron Donating/Withdrawing Strength ^1^	Steric Bulk ^2^
*Electron Donating Groups*		
-OH (hydroxyl)	Strong	Low
-R (Alkyl)	Medium	Medium (<3 C), high (≥3 C) *
-NH_2_, -NHR, -NR_2_ (amine)	Strong	Medium
-ROR (ether)	Medium	Low
-SCH_3_	Weak	Low
*Electron Withdrawing Groups*		
-COOH (carboxyl)	Medium	Medium
-C_6_H_5_ (phenyl)	Weak	High
-F, -Br, -Cl (Halogen)	Medium	Low
-CONH_2_, -CONHR, -CONR_2_ (amide)	Strong	Medium
-COOR (ester)	Medium	Medium
-COR (acyl)	Medium	Medium
-SO_2_OH	Medium	Unknown
-SO_2_NHR	Strong	Unknown

^1^ source: [30]; ^2^ source: [31,32]; * refers to the number of carbon of the substituent.

## Appendix A

**Table A1:** Representative OCs shortlisted for susceptibility assessment and their respective assessment criteria data.

Organic Contaminant	Chemical Formula	Chemical Structure ^ℇ^	Molecular Weight (g/mol)	EDG Score	EWG Score	Net Electron Donating Group Density	pKa	Log K_ow_	Steric Bulk
Sulfamethoxazole	C_10_H_11_N_3_O_3_S		253.28	5	3	0.79	5.81 ^1^	0.89 ^2^	Medium
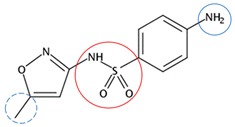
Erythromycin	C_37_H_67_NO_13_		733.93	19	4	2.04	8.90 ^3^	3.10 ^3^	Medium
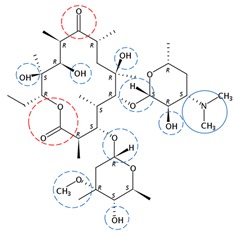
Tetracycline	C_22_H_24_N_2_O_8_		444.43	13	6	1.58	9.69 ^4^	−1.33 ^5^	Medium
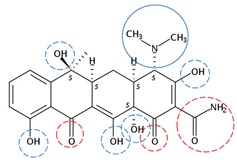
Ofloxacin	C_18_H_20_FN_3_O_4_		361.37	8	6	0.55	7.65 ^6^	0.00 ^5^	Low
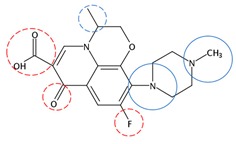
Trimethoprim	C_14_H_18_N_4_O_3_		290.32	12	0	4.13	7.20 ^7^	0.91 ^2^	Medium
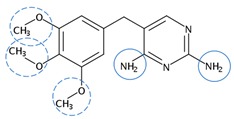
Diclofenac	C_14_H_11_Cl_2_NO_2_		296.15	3	6	−1.01	4.20 ^2^	0.70 ^2^	Medium
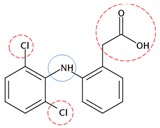
Carbamazepine	C_15_H_12_N_2_O		236.27	0	2	-0.85	13.9 ^8^	2.25 ^5^	Medium
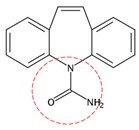
Metoprolol	C_15_H_25_NO_3_		267.36	11	0	4.11	9.60 ^2^	2.15 ^2^	High
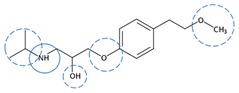
Gemfibrozil	C_15_H_22_O_3_		250.33	8	2	2.40	4.70 ^2^	4.77 ^2^	High
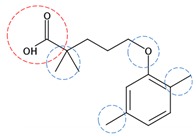
Benzophenone-3	C_14_H_12_O_3_		228.24	5	3	0.88	7.10	3.79 ^9^	Medium
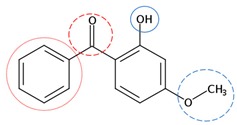
Fluoxetine	C_17_H_18_F_3_NO		309.33	5	3	0.65	8.70 ^10^	4.05 ^10^	High
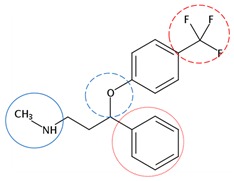
Oestrone	C_18_H_22_O_2_		270.37	3	2	0.74	10.34 ^2^	3.13 ^2^	Low
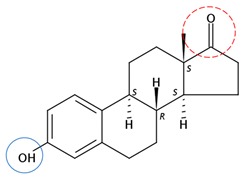
Galaxolide	C_18_H_26_O		258.40	12	0	4.64	/	5.90 ^11^	Medium
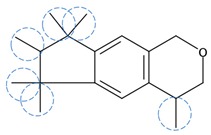
Triclosan	C_12_H_7_Cl_3_O_2_		289.54	5	6	−0.35	7.80 ^10^	4.76 ^10^	Low
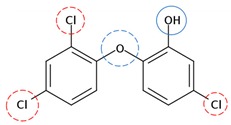
BDE-209	C_12_Br_10_O		959.17	2	20	−1.88	/	12.11 ^12^	Low
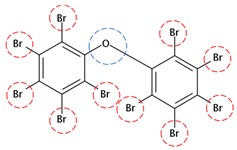
PBEB	C_8_H_5_Br_5_		500.65	2	10	−1.60	/	/	Medium
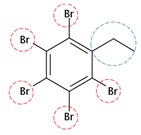
TDCPP	C_9_H_15_Cl_6_O_4_P		430.90	/	/	/	/	3.76 ^9^	High
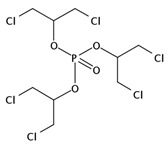
DEHP	C_24_H_38_O_4_		390.56	8	4	1.02	/	7.60 ^12^	High
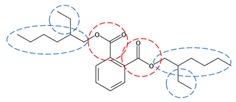
OBT	C_7_H_5_NOS		151.19	0	2	−1.32	/	1.76 ^11^	Medium
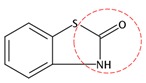
TBT	C_12_H_27_Sn		290.05	/	/	/	/	3.52 ^11^	High
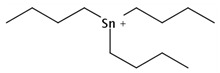
PFOA	C_8_HF_15_O_2_		414.07	/	/	/	1.30 ^13^	4.81 ^9^	Medium
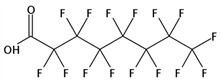
PFOS	C_8_F_17_O_3_S		499.12	/	/	/	−3.3 ^9^	4.49 ^9^	Medium
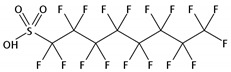
TCDD	C_12_H_4_Cl_4_O_2_		321.97	4	8	−1.24	/	6.80 ^9^	Low
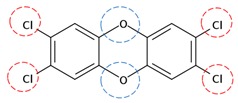
2,3,6,7-PCN	C_10_H_4_Cl_4_		265.95	0	8	-3.01	/	6.19 ^9^	Low
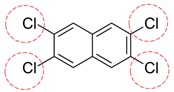
CP-10	C_10_H_16_Cl_6_		348.98	/	/	/	/	/	Medium
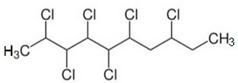

^1^ source: [57], ^2^ source: [58], ^3^ source: [59], ^4^ source: [60], ^5^ source: [61], ^6^ source: [62], ^7^ source: [63] ^8^ source: [29], ^9^ source: [64], ^10^ source: [65], ^11^ source: [66], ^12^ source: [67], ^13^ source: [68].

Legend:

Red rings		Electron withdrawing groups
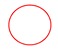
Blue rings		Electron donating groups
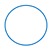
Solid rings		Strong groups
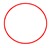
Thick dashed rings		Encircle medium groups
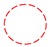
Thin dashed rings		Weak groups
